# Enzyme Inhibitory Kinetics and Molecular Docking Studies of Halo-Substituted Mixed Ester/Amide-Based Derivatives as Jack Bean Urease Inhibitors

**DOI:** 10.1155/2020/8867407

**Published:** 2020-12-24

**Authors:** Muhammad Rashid, Hummera Rafique, Sadia Roshan, Shazia Shamas, Zafar Iqbal, Zaman Ashraf, Qamar Abbas, Mubashir Hassan, Zia Ur Rahman Qureshi, Muhammad Hassham Hassan Bin Asad

**Affiliations:** ^1^Department of Chemistry, Allama Iqbal Open University, Islamabad 44000, Pakistan; ^2^Department of Chemistry, University of Gujrat, Gujrat 50700, Pakistan; ^3^Department of Zoology, University of Gujrat, Gujrat 50700, Pakistan; ^4^Department of Physiology, University of Sindh, Jamshoro, Pakistan; ^5^Institute of Molecular Biology and Biotechnology, The University of Lahore, Lahore, Pakistan; ^6^Department of Pharmacy, SBK Women University, Quetta, Balochistan, Pakistan; ^7^Department of Pharmacy, COMSATS University Islamabad, Abbottabad Campus, Pakistan; ^8^Institute of Fundamental Medicine, Department of Genetics, Kazan Federal University, Russia

## Abstract

A series of halo-substituted mixed ester/amide-based analogues **4a-l** have been prepared as jack bean urease inhibitor, which showed good to excellent inhibition of enzyme activity. The role of halo-substituted benzoyl moieties and alkyl substituted anilines in urease inhibitory kinetics was also investigated. The alkyl-substituted anilines **1a–b** reacted with chloroacetyl chloride to afford intermediates **2a-b**, which were then reacted with different halo-substituted benzoic acids **3a–f** to prepare the title compounds **4a-l**. The chemical structures of final products **4a-l** were ascertained by FTIR, ^1^H NMR, ^13^C NMR, and mass spectra. The compound **4b** showed remarkable activity with IC_50_1.6 ± 0.2 nM, better than the standard thiourea having IC_50_472.1 ± 135.1 nM. The 2-chloro-substituted phenyl ring on one side of compound **4b** and 4-isopropyl-substituted benzene on the other side play an essential role in inhibition of urease activity. Lineweaver–Burk plots (kinetics study) indicated about **4b** derivative as a mixed type of inhibitor. The virtual screening performed against urease enzyme (PDBID 4H9M) showed that compounds **4b** and **4e** have binding energies of −7.8 and −7.9 Kcal/mol, respectively. Based upon our results, it was found that derivative **4b** is a highly potent urease inhibitor, better than the standard thiourea.

## 1. Introduction

**Figure 1:**
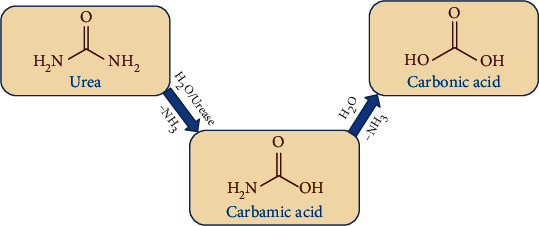
Hydrolysis of urea by urease enzyme.

Urease (EC.3.5.1.5) is an enzyme of the amidohydrolase and phosphotriesterase family with nickel atoms present in their active binding sites. They catalyze hydrolysis of urea into carbamic acids which then cleaved into carbon dioxide and ammonia (Figure 1) [1–4] which in turn increased the pH. The increased level of urease enzyme is also associated with serious health problems like stomach cancer, peptic ulceration, and pyelonephritis [5–7].

The bacterial ureases increase the rate of urea hydrolysis which is associated with different biological disorders like gastric cancer, urinary tract infections, liver cirrhosis, liver inflammation, and hepatic coma [8]. The *Helicobacter pylori* and other bacteria live at lower pH of the stomach by creating a shielding ammonium cloud during colonization [9–14]. It is statistically calculated that nearly half of the world's population suffer due to *Helicobacter pylori* [15–18]. Some other bacteria, mainly *Proteus*, *Klebsiella*, *Pseudomonas*, and *Staphylococcus* species, lead to kidney stones, often called infectious stones, such as struvite (magnesium ammonium phosphate) [NH_4_MgPO_4_.6H_2_O)] and carbonate apatite [Ca_10_(PO_4_)_6_CO_3_] [19, 20]. The congenital problems associated with a well-known urease inhibitor hydroxamic acids limit its clinical utility. The need of new antibiotics is also necessary due to the emergence of resistance in *Helicobacter pylori* against metronidazole, clarithromycin, and levofloxacin [21, 22].

The already reported urease inhibitors are urea derivatives, hydroxamic acids, heavy metal ions, quinones, polyphenols, and organosulfur compounds. Thiourea and hydroxyurea bind with the active binding site of target enzyme in the same way as urea binds with urease. It has been studied that amides and hydroxyl-substituted acids display antimicrobial activity [23]. It is expected that the mixed ester and amide functionalities may assist in designing more potent urease inhibitors. Our previous work focusses on drug development through enzyme-based assay [24, 25]. Hence, the compounds **4a–l** were designed and synthesized having ester amide linkages along with halo-substituted benzoyl moiety for their improved urease inhibitory activity by creating secondary interactions with the protein part of the enzyme.

## 2. Materials and Methods

### 2.1. Chemistry

Reagents and chemicals were used as received without further purification. A digital Gallen Kamp (SANYO) apparatus was used to record the melting points. The Perkin Elmer spectrophotometer was used to record FTIR spectra by using the KBr pellet technique and is expressed in centimeters. The ^1^H NMR (300 MHz) and ^13^C NMR (75 MHz) (*δ*-ppm) analyses were done using a Bruker AM-300 spectrometer. The mass spectra were recorded on a GC–MS spectrometer by using the 6890 Network GC system (Agilent Technologies).

#### 2.1.1. Synthesis of Compounds **4a–l**

In the first step, **1a** (4-isopropyl aniline) and **1b** (2-methyl-5-isopropyl aniline) were reacted with chloroacetyl chloride to form **2a** and **2b**, respectively. In the second step, **2a** and **2b** were also made to react with different halo-substituted benzoic acids **3a–f** to form **4a–l**, the final products.

#### 2.1.2. Synthesis of Chloroacetyl Alkylated Aniline Derivative **2a–b**

4-Isopropylaniline **1a** (0.3 g, 0.01 mol) and 5-isopropyl-2-methylaniline **1b** (0.3 g, 0.01 mol) were mixed in a reaction flask; then, triethylamine (0.01 mol) was added in dichloromethane (30 mL). To this reaction mixture, 0.01 mol of chloroacetyl chloride was slowly added with constant stirring for 1 h at 0–5°C. After completion of the reaction, mixture was washed by 1% HCl (2 × 50.0 mL), 1% sodium hydroxide solution (2 × 50.0 mL), and, finally, 1% sodium chloride solution (2 × 50.0 mL) and then dried by magnesium sulfate to get the intermediate products **2a–b**. These products were purified by silica gel column chromatography by using *n*-hexane and ethyl acetate as mobile phase.

#### 2.1.3. Synthesis of Compounds **4a–l**

**Scheme 1:**
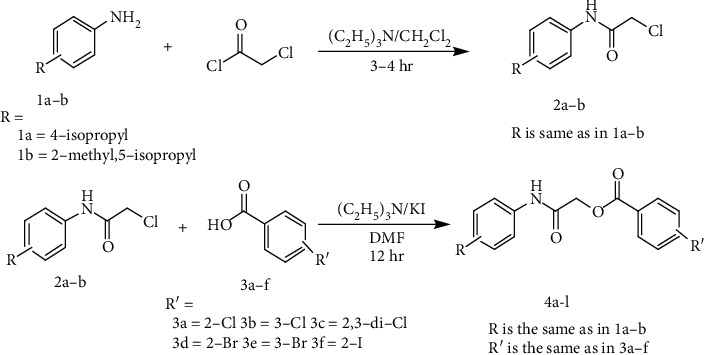
Synthesis of compounds **4a–l.**

The intermediates **2a–b** (0.01 mol) were reacted with 0.01 mol of different halogen-substituted benzoic acids **3a–f** in the presence of equimolar amounts of triethylamine and KI in 10 mL DMF; mixture was stirred at 25°C for 8–10 h. The mixture was poured into ice-cold water, and product was extracted in ethyl acetate (3 × 25 mL). The product was washed with 1% HCl (2 × 50.0 mL), 1% NaOH solution (2 × 50.0 mL), and 1% NaCl solution (2 × 50 mL), and then, crude products were purified by silica gel column chromatography (Scheme 1).

#### 2.1.4. 2-((4-Isopropylphenyl)amino)-2-oxoethyl 2-Chlorobenzoate **4a**

Yield: 60%; m.p 94–96°C; *R*_f_ = 0.38 (C_6_H_14_/CH_3_CO_2_C_2_H_5_ 3:1); FTIR *ν*_max_ cm^−1^: 3316 (N–H), 2965 (sp^3^ C–H aromatic), 2850 (C–H saturated), 1742 (C=O aromatic ester), 1685 (C=O amide), 1588 (C=C aromatic), 1237 (C–O ester), 746 (C–Cl); ^1^H NMR (CDCl_3_): 7.99 (1H, s, N–H), 7.58 (d, *J* = 4.2 Hz, 1H), 7.54 (1H, m,), 7.49 (2H, d, *J* = 8.4 Hz), 7.42 (1H, m), 7.23 (2H, d, *J* = 8.5 Hz), 4.98 (2H, s, methylene), 2.90 (1H, sep), 1.25 (6H, d, *J* = 6.9 Hz alkyl); ^13^C NMR: 164.53 (C=O aromatic ester), 164.32 (C=O amide), 145.79, 134.46, 133.63, 133.17, 132.57, 131.33, 128.90, 127.27, 127.06, 120.18 (Aromatic), 64.20, 33.65, 24.02 (Aliphatic); EI-MS (*m*/*z*, %): 331 (M^+^, 40), 318 (14), 296 (0.6), 258 (1.1), 197 (6), 139 (100), 136 (56), 119(64), 111 (36), 91 (21), 75 (18), 50 (8).

#### 2.1.5. 2-((4-Isopropylphenyl)amino)-2-oxoethyl 3-Chlorobenzoate **4b**

Yield: 68%; m.p 96–98°C; *R*_f_ = 0.41 (C_6_H_14_/CH_3_CO_2_C_2_H_5_ 3:1); FTIR *ν*_max_ cm^−1^: 3257 (N–H), 3117 (unsaturated C-H), 2963 (saturated C–H), 1739 (C=O aromatic ester), 1670 (C=O amide), 1537 (C=C aromatic), 1275 (C–O ester), 759 (C–Cl); ^1^H NMR (CDCl_3_): 8.18 (1H, s), 8.01 (1H, dd, *J*_1_ = 5.4 Hz, *J*_2_ = 1.2 Hz), 7.72 (1H, s, N–H), 7.64 (1H, dd, *J*_1_ = 7.3 Hz, *J*_2_ = 0.9 Hz), 7.49 (2H, d, *J* = 7.8 Hz), 7.44 (1H, m), 7.23 (2H, d, *J* = 8.4 Hz), 4.97 (2H, s), 2.91 (1H, sep, *J* = 6.9 Hz), 1.25 (6H, d, *J* = 6.9 Hz); ^13^C NMR: 164.68 (C=O aromatic ester), 164.17 (C=O amide), 145.99, 134.99, 134.18, 133.90, 130.60, 130.09, 129.89, 127.94, 127.05, 120.55 (Aromatic), 63.95, 33.64, 23.98 (Aliphatic); EI-MS (*m*/*z*, %): 331 (M^+^, 40), 318 (10), 258 (1.1) 197 (9), 139 (100), 136 (74), 119 (88), 111 (58), 91 (32), 75 (23), 50 (8).

#### 2.1.6. 2-((4-Isopropylphenyl)amino)-2-oxoethyl 3,4-Dichlorobenzoate **4c**

Yield: 65%; m.p 107–108°C; *R*_f_ = 0.41 (C_6_H_14_/CH_3_CO_2_C_2_H_5_ 3:1); FTIR *ν*_max_ cm^−1^: 3279 (N–H stretch), 3066 (unsaturated C–H), 2962 (saturated C–H), 1724 (C=O aromatic ester), 1658 (C=O amide), 1541 (C=C aromatic), 1232 (C–O ester), 782 (C–Cl); ^1^H NMR (DMSO): 10.11 (1H, s, N–H), 8.16 (1H, s), 7.94 (1H, d, *J* = 8 Hz), 7.84 (1H, d, *J* = 8 Hz), 7.46 (2H, d, *J* = 8 Hz), 7.15 (2H, d, *J* = 9 Hz), 4.91 (2H, s), 2.83 (1H, sep, *J* = 8 Hz), 1.14 (6H, d, *J* = 8 Hz); ^13^C NMR: 165.17 (C=O aromatic ester), 164.08 (C=O amide), 144.16, 137.05, 136.52, 132.24, 131.78, 130.17, 129.90, 126.95, 119.93, 110.97 (Aromatic), 64.03, 33.30, 24.30 (Aliphatic); EI-MS (*m*/*z*, %): 365 (M^+^, 37), 350 (20), 203 (4), 173 (100), 145 (44), 119 (98), 135 (68), 157 (33), 91 (42), 65 (6).

#### 2.1.7. 2-((4-Isopropylphenyl)amino)-2-oxoethyl 2-Bromobenzoate **4d**

Yield: 70%; m.p 89–90°C; *R*_f_ = 0.52 (C_6_H_14_/CH_3_CO_2_C_2_H_5_ 3:1); FTIR *ν*_max_ cm^−1^: 3307 (N–H), 2922 (unsaturated C–H), 2853 (saturated C–H), 1742 (C=O aromatic ester), 1685 (C=O amide), 1537 (C=C aromatic), 1106 (C–O ester), 743 (C–Br); ^1^H NMR (CDCl_3_): 8.30 (1H, s, N–H), 7.93 (1H, m), 7.75 (1H, m), 7.52 (2H, d, *J* = 8.4 Hz), 7.47 (1H, m), 7.45 (1H, m), 7.23 (2H, d, *J* = 8.5 Hz), 4.98 (2H, s), 2.91 (1H, sep, *J* = 6.2 Hz), 1.25 (6H, d, *J* = 9.0 Hz); ^13^C NMR: 164.79 (C=O ester), 164.51 (C=O amide), 145.81, 134.51, 134.44, 133.54, 132.41, 131.19, 127.76, 127.06, 121.18, 120.26 (Aromatic), 64.27, 33.65, 24.02 (Aliphatic); EI-MS (*m*/*z*, %): 375 (M^+^, 50), 355 (16), 243 (12), 183 (100), 157 (33), 135 (90), 119 (64), 91 (48), 75 (32), 50 (14).

#### 2.1.8. 2-((4-Isopropylphenyl)amino)-2-oxoethyl 3-Bromobenzoate **4e**

Yield: 70%; m.p 91–92°C; *R*_f_ = 0.50 (C_6_H_14_/CH_3_CO_2_C_2_H_5_ 3:1); FTIR *ѵ*_max_ cm^−1^: 3256 (N–H), 2964 (Ar–H), 2877 (sp^3^ C–H), 1739 (C=O aromatic ester), 1669 (C=O amide), 1536 (C=C aromatic), 1250 (C–O ester), 729 (C–Br); ^1^H NMR (CDCl_3_): 8.26 (1H, s), 8.07 (1H, dd, *J*_1_ = 7.8 Hz, *J*_2_ = 1.2 Hz), 7.81 (1H, dd, *J*_1_ = 8.1 Hz, *J*_2_ = 0.9 Hz), 7.70 (1H, s, N–H), 7.47 (2H, d, *J* = 8.4 Hz), 7.40 (1H, m), 7.23 (2H, d, *J* = 8.4 Hz), 4.99 (2H, s), 2.91 (1H, sep, *J* = 6.9 Hz), 1.25 (6H, d, *J* = 6.9 Hz); ^13^C NMR: 165.28 (C=O aromatic ester), 164.60 (C=O amide), 144.13, 136.78, 136.56, 132.25, 13.60, 130.09, 131.89, 131.58, 128.89, 126.95, 122.29, 119.90 (Aromatic), 63.89, 33.30, 24.37 (Aliphatic); EI-MS (*m*/*z*, %): 375 (M^+^, 38), 355 (16), 243 (12), 157 (33), 135 (100), 119 (98), 91 (48), 75 (32), 50 (14).

#### 2.1.9. 2-((4-Isopropylphenyl)amino)-2-oxoethyl 2-Iodobenzoate **4f**

Yield: 70%; m.p 91–92°C; *R*_f_ = 0.50 (C_6_H_14_/CH_3_CO_2_C_2_H_5_ 3:1); FTIR *ν*_max_ cm^−1^: 3269 (N–H stretch), 3133 (Ar–H), 2953 (sp^3^ C–H), 1734 (C=O aromatic ester), 1671 (C=O amide), 1583 (C=C aromatic), 1241 (C–O ester), 741 (C–I); ^1^H NMR (DMSO): 10.03 (1H, s, N–H), 8.03 (1H, d, *J* = 8), 7.87 (1H, m), 7.70 (1H, m) 7.54 (1H, m), 7.47 (2H, d, *J* = 8 Hz), 7.16 (2H, d, *J* = 8 Hz), 4.88 (2H, s), 2.80 (1H, sep, *J* = 8 Hz), 1.92 (6H, d, *J* = 9.0 Hz); ^13^C NMR: 166.20 (C=O aromatic ester), 165.23 (C=O amide), 144.90, 141.35, 136.61, 134.91, 133.83, 132.02, 131.45, 129.09, 128.73, 126.96 ((Aromatic)), 63.84, 33.30, 24.38 (Aliphatic); EI-MS (*m*/*z*, %): 423 (M^+^, 64), 289 (8), 231 (100), 261 (6), 203 (38), 146 (9), 135 (52), 119 (64), 91 (46), 75 (35), 50 (12).

#### 2.1.10. 2-((5-Isopropyl-2-methyl phenyl) Amino)-2-oxoethyl 2-Chloro Benzoate **4g**

Yield: 65%; m.p 115–116°C; *R*_f_ = 0.48 (C_6_H_14_/CH_3_CO_2_C_2_H_5_ 3:1); FTIR *ν*_max_ cm^−1^: 3256 (N–H), 2990 (unsaturated C-H), 2878 (saturated C–H), 1745 (C=O aromatic ester), 1685 (C=O amide), 1541 (C=C aromatic), 1124 (C–O ester), 747 (C–Cl); ^1^H NMR (CDCl_3_): 8.02 (1H, s, N–H), 7.99 (1H, d, *J* = 8.4 Hz), 7.72 (1H, s), 7.55 (1H, d, *J* = 4.5 Hz), 7.53 (1H, m), 7.48 (1H, m), 7.15 (1H, d, *J* = 7.8 Hz), 7.02 (1H, d, *J* = 6.3 Hz), 5.02 (2H, s), 2.91 (1H, sep, *J* = 10.2 Hz), 2.24 (3H, s), 1.25 (6H, d, *J* = 8.5 Hz); ^13^C NMR: 164.80 (C=O aromatic ester), 164.38 (C=O amide), 147.48, 133.60, 132.24, 131.41, 131.36, 130.91, 130.53, 128.82, 128.80, 127.13, 123.92, 121.67 (Aromatic), 64.34, 33.79, 23.99, 17.39 (Aliphatic); EI-MS (*m*/*z*, %): 345 (M^+^, 64), 206 (8), 176 (9), 148 (54), 139 (100), 111 (26), 75 (11), 50 (2).

#### 2.1.11. 2-((5-Isopropyl-2-ethylphenyl) Amino)-2-oxoethyl 3-Chlorobenzoate **4h**

Yield: 76%; m.p 119–120°C; *R*_f_ = 0.54 (C_6_H_14_/CH_3_CO_2_C_2_H_5_ 3:1); FTIR *ν*_max_ cm^−1^: 3258 (N–H), 2957 (unsaturated C–H), 2869 (saturated C–H), 1727 (C=O aromatic ester), 1666 (C=O amide), 1575 (C=C aromatic), 1249 (C–O ester), 756 (C–Cl); ^1^H NMR (CDCl_3_): 8.11 (1H, s), 8.03 (1H, d, *J* = 7.8 Hz), 7.83 (1H, s), 7.75 (1H, s), 7.64 (1H, d, *J* = 8.4 Hz), 7.48 (1H, m), 7.15 (1H, d, *J* = 7.8 Hz), 7.03 (1H, d, *J* = 8.1 Hz), 5.02 (2H, s), 2.92 (1H, sep, *J* = 6.9 Hz), 2.25 (3H, s), 1.25 (6H, d, *J* = 6.9 Hz); ^13^C NMR: 165.64 (C=O aromatic ester), 164.68 (C=O amide), 146.48, 135.76, 133.93, 133.88, 131.72, 131.28, 130.66, 129.94, 129.45, 128.59, 124.04, 123.51 (Aromatic), 63.91, 33.40, 24.32, 17.28 (Aliphatic); EI-MS (*m*/*z*, %): 345 (M^+^, 12), 206 (8), 176 (100), 148 (8), 139 (76), 119 (8), 75 (20), 50 (6).

#### 2.1.12. 2-((5-Isopropyl-2-methyl phenyl) Amino)-2-oxoethyl 2-Bromobenzoate **4i**

Yield: 68%; m.p 112–113°C; *R*_f_ = 0.43 (C_6_H_14_/CH_3_CO_2_C_2_H_5_ 3:1); FTIR *ν*_max_ cm^−1^: 3257 (N–H), 2958 (unsaturated C–H), 2870 (saturated C–H), 1741 (C=O aromatic ester), 1666 (C=O amide), 1541 (C=C aromatic), 1242 (C–O ester), 743 (C–Br); ^1^H NMR (CDCl_3_): 8.02 (1H, s, N–H), 7.99 (1H, dd, *J*_1_ = 6.3 Hz, *J*_2_ = 2.7 Hz), 7.75 (1H, dd, *J*_1_ = 7.2 Hz, *J*_2_ = 2.7 Hz), 7.47 (1H, m), 7.43 (1H, m), 7.15 (1H, d, *J* = 7.5 Hz), 7.03 (1H, d, *J* = 8.5 Hz), 5.01 (2H, s), 2.91 (1H, sep, *J* = 7.5 Hz), 2.24 (3H, s), 1.25 (6H, d, *J* = 7.2 Hz); ^13^C NMR: 165.53 (C=O aromatic ester), 164.07 (C=O amide), 146.70, 137.02, 135.73, 132.19, 131.71, 131.59, 130.67, 130.17, 129.96, 124.09, 123.56, 110.94 (Aromatic), 64.05, 33.40, 24.32, 17.81 (Aliphatic); EI-MS (*m*/*z*, %): 391 (M^+^, 35), 216 (16), 241 (6), 148 (96), 105 (24), 76 (23), 50 (10).

#### 2.1.13. 2-((5-Isopropyl-2-methyl phenyl) Amino)-2-oxoethyl 3-Bromobenzoate **4j**

Yield: 72%; m.p 116-118°C; *R*_f_ = 0.62 (C_6_H_14_/CH_3_CO_2_C_2_H_5_ 3:1); FTIR *ν*_max_ cm^−1^: 3254 (N–H), 2956 (unsaturated C–H), 2922 (saturated C–H), 1735 (C=O ester), 1667 (C=O amide), 1569 (Ar–C=C), 1224 (C–O ester), 740 (C–Br); ^1^H NMR (CDCl_3_): 8.27 (1H, s,), 8.06 (1H, dd, *J*_1_ = 7.8 Hz, *J*_2_ = 0.9 Hz), 7.83 (1H, dd, *J*_1_ = 6.6 Hz, *J*_2_ = 0.9 Hz), 7.78 (1H, s), 7.76 (1H, s, N–H), 7.42 (1H, m), 7.15 (1H, d, *J* = 7.8 Hz), 7.02 (1H, d, *J* = 7.5 Hz), 5.01 (2H, s), 2.92 (1H, sep, *J* = 6.9 Hz), 2.25 (3H, s), 1.26 (6H, d, *J* = 6.9 Hz); ^13^C NMR: 165.56 (C=O aromatic ester), 165.37 (C=O amide), 146.68, 135.79, 134.59, 134.01, 132.05, 131.70, 130.68, 129.77, 128.29, 123.95, 123.34, 121.14 (Aromatic), 63.86, 33.41, 24.33, 17.85 (Aliphatic); EI-MS (*m*/*z*, %): 389 (M^+^, 26), 216 (16), 241 (6), 149 (100), 148 (96), 105 (24), 76 (23), 50 (10).

#### 2.1.14. 2-((5-Isopropyl-2-methyl phenyl) Amino)-2-oxoethyl 3,4-Dichlorobenzoate **4k**

Yield: 76%; m.p 115–116°C; *R*_f_ = 0.38 (C_6_H_14_/CH_3_CO_2_C_2_H_5_ 3:1); FTIR *ν*_max_ cm^−1^: 3264 (N–H), 2926 (unsaturated C–H), 2850 (saturated C–H), 1741 (C=O aromatic ester), 1667 (C=O amide), 1557 (C=C aromatic), 1240 (C–O ester), 768 (C–Cl); ^1^H NMR (DMSO): 9.54 (1H, s, N–H), 8.16 (1H, s), 8.04 (1H, d, *J* = 9), 7.90 (1H, d, *J* = 8 Hz), 7.52 (1H, d, *J* = 8 Hz), 7.23 (1H, s), 7.12 (1H, d, *J* = 8 Hz), 4.95 (2H, s), 2.82 (1H, sep, *J* = 8 Hz), 2.14 (3H, s), 1.15 (6H, d, *J* = 4 Hz); ^13^C NMR: 165.64 (C=O aromatic ester), 164.58 (C=O amide), 146.68, 136.75, 135.76, 132.33, 131.90, 131.50, 130.66, 129.92, 128.94, 124.03, 123.49, 122.26 (Aromatic), 63.91, 33.40, 24.32, 17.28 (Aliphatic); EI-MS (*m*/*z*, %): 379 (M^+^, 29), 231 (8), 206 (9), 173 (100), 149 (88), 119 (7), 135 (68), 157 (33), 91 (7), 65 (5).

#### 2.1.15. 2-((5-Isopropyl-2-methyl phenyl) Amino)-2-oxoethyl 2-Iodobenzoate **4l**

Yield: 72%; m.p 99–101°C; *R*_f_ = 0.69 (C_6_H_14_/CH_3_CO_2_C_2_H_5_ 3:1); FTIR *ν*_max_ cm^−1^: 3256 (N–H), 2957 (unsaturated C–H), 2921 (saturated C–H), 1740 (C=O aromatic ester), 1663 (C=O amide), 1569 (C=C aromatic), 1221 (C–O ester), 739 (C–I); ^1^H NMR (DMSO): 9.54 (1H, s, N–H), 8.03 (1H, d, *J* = 8 Hz), 7.89 (1H, d, *J* = 8 Hz), 7.54 (1H, m), 7.30 (1H, m), 7.25 (1H, s), 7.10 (1H, d, *J* = 8 Hz), 6.96 (1H, d, *J* = 8 Hz), 4.92 (2H, s), 2.76 (1H, sep, *J* = 8 Hz), 2.15 (3H, s), 1.14 (6H, d, *J* = 8 Hz); ^13^C NMR: 166.12 (C=O aromatic ester), 165.60 (C=O amide), 146.68, 141.37, 135.80, 134.82, 133.84, 131.53, 130.68, 129.78, 128.69, 123.35, 110.96 (Aromatic), 63.58, 33.41, 24.34, 17.98 (Aliphatic); EI-MS (*m*/*z*, %): 423 (M^+^, 47), 289 (5), 261 (2), 231 (100), 203 (29), 176 (10), 149 (46), 105 (23), 76 (31), 50 (8).

### 2.2. Jack Bean Urease Inhibition Assay

The inhibitory effects on urease activity were done based on the Weatherburn method, using the indophenol scheme by determining the amount of ammonia produced [26]. Briefly, 20 *μ*L of jack bean urease enzyme (0.135 units) and 20 *μ*L of the tested compounds were taken in 50 *μ*L buffer and were incubated for 30 min. Temperature was kept at 37°C in a 96-well plate reader. The buffer solution was made by 0.01 molar K_2_HPO_4_/KH_2_PO_4_, 1 mM EDTA, 100 mM urea, and 0.01 M LiCl; pH was maintained at 7.0. Then, 50 *μ*L of phenol reagent (1% *w*/*v* phenol and 0.005% *w*/*v* sodium nitroprusside) was added in 50 *μ*L of alkali reagent in each well. The alkali reagent was prepared by 0.5% *w*/*v* NaOH and 0.1% NaOCl. Absorbance was measured at 625 nm using OPTI_Max_, a tunable microplate reader, after 10 min interval in a triplicate manner. The thiourea was used as a standard jack bean urease inhibitor. The data has been analyzed using software, GraphPad Prism, and statistical analysis (SD) was performed by using SigmaPlot software to get SD values of the bioassay results.

### 2.3. Molecular Docking Studies

The Protein Data Bank (PDB) was used to retrieve the structure of the jack bean urease enzyme PDBID 4H9M [27]. The University of California, San Francisco (UCSF) Chimera 1.10.1 tool was used to save the crystal structure of the enzyme in its stable conformation [28]. The MolProbity server (http://molprobity.biochem.duke.edu/) was used to calculate the Ramachandran plot values and the stereochemical properties of the urease structure [29] and ProtParam [30]. Hydrophobicity graphs of the targeted protein were generated using Discovery Studio 4.1 client tool (BIOVIA 5005 Wateridge Vista Drive, San Diego, CA 92121, USA) [31]. VANDAR 1.8, the online server, was used to predict protein architecture statistical percentage of receptor protein helices, beta sheets, coils, and turns [32]. The UCSF Chimera 1.10.1 tool was used to save the stable structures of the synthesized compounds (**4a–l**), which were electronically sketched using the ACD/Chem sketch tool. The molecular docking experiment was performed by the PyRx docking tool. Before going for the docking experiment, the active site of the target protein was analyzed from the PDB and was also compared with literature data [33]. The grid was generated based on binding pocket residues with appropriate coordinate values in *XYZ* dimensions, respectively. The center values of the grid box were set as center *X*_ = 1.48, center_*Y* = −55.22, and center_*Z* = −26.48. The size values of grid box were adjusted accordingly as *X* = 66.60, *Y* = 60.08, and *Z* = 56.84. Exhaustiveness value = 8 was set as default and was used to maximize the conformational analysis of binding. Each of the synthesized ligands was docked separately against the urease enzyme to predict the binding affinity. The predicted energies were compared on the lowest energies and were used to generate structure–activity relationship. Discovery Studio 2.1.0 was used to depict three-dimensional graphs for all of the docked ligand–protein complexes. The AutoDock tool has been used for determination of inhibition constant (*K*_i_) for the most potent derivative **4b**.

## 3. Results

### 3.1. Chemistry

The halo-substituted mixed ester/amide derivatives **4a-l** were successfully synthesized by following the simple reaction route in good yields. The final products **4a-l** were prepared by using our previously developed method with minor modifications [34]. Chloroacetyl chloride was reacted with alkyl-substituted anilines in the presence of dichloromethane as a solvent (Scheme 1). In this reaction, the amino group in compounds **1a–b** displaced the chlorine atom attached with carbonyl carbon of the chloroacetyl chloride to give chloroacetyl derivatives **2a–b**. According to the FTIR spectrum, compounds **4a–l** showed a characteristic peak of amide carbonyl (C=O) at 1640–1685 cm^−1^, ester (C=O) at 1724–1745 cm^−1^, and secondary (N–H) stretching at 3250–3307 cm^−1^. The amide carbonyl absorption appeared at 1670 cm^−1^ in **2a** and 1655 cm^−1^ in **2b** and secondary (N–H) absorption at 3270 cm^−1^ in **2a** and 3257 cm^−1^ in **2b** in the FTIR spectrum, which confirmed the formation of **2a–b**. The final products **4a–l** were synthesized by nucleophilic substitution of chloroacetyl derivative **2a–b** by halo-substituted benzoic acids **4a–f**.

### 3.2. Biological Evaluation

**Figure 2:**
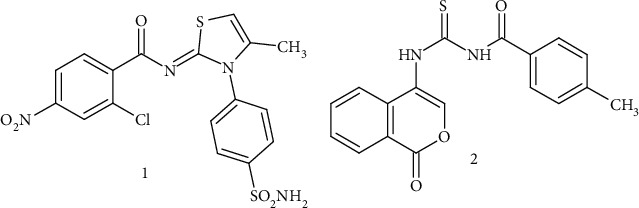
Structures of already reported urease inhibitors.

The compounds **4a–l** having ester and amide linkages were designed and synthesized as jack bean urease inhibitors. We have already synthesized some heterocyclic derivatives as jack bean urease inhibitors. The iminothiazoline-sulfonamide **1** and 4-aminocoumarine thiourea derivative **2 (**Figure 2**)** showed good activity with IC_50_ values 58 nM and 6.5 nM, respectively [35, 36]. These derivatives possess iminothiazoline bearing sulfonamide moieties and thiourea bearing coumarin ring system. These derivatives exhibited good urease inhibitory activity especially compound **2** comparable to **4b** reported in present studies.

In the present work, halo-substituted benzoic acid moiety was attached to create secondary interactions with amino acid residues of urease enzyme, and we obtained excellent activity compared to standard thiourea. The derivatives **4a–l** exhibited good to excellent urease inhibitory activity, compared to thiourea used as positive control. The bioassay results and mean standard errors *n* = 3 are presented in Table 1.

### 3.3. Kinetic Study

**Figure 3:**
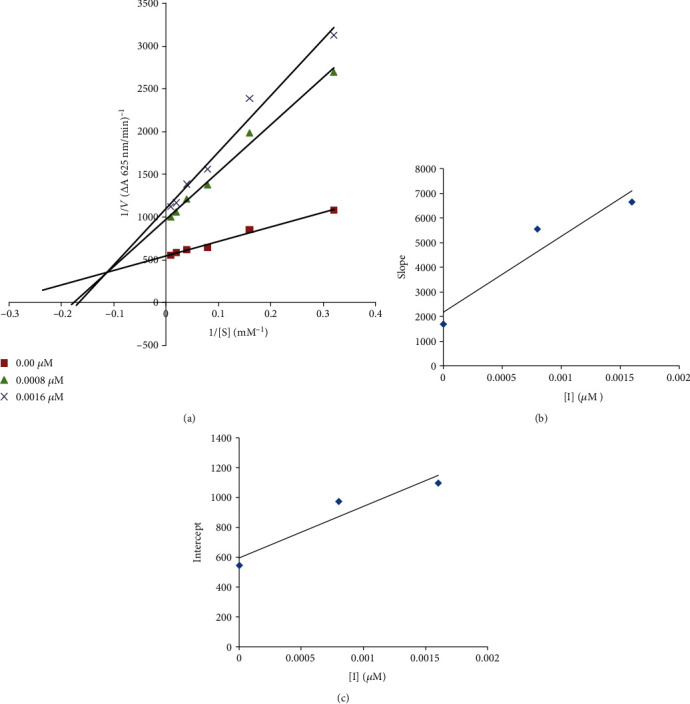
Enzyme inhibitory kinetic mechanism of the most potent derivative **4b** by Lineweaver–Burk plots. 1/*V*_max_: reciprocal of maximum velocity; 1/[S]: reciprocal of substrate concentration.

The derivative **4b** was selected on the basis of its high activity for the determination of its kinetic mechanism on jack bean urease. The enzyme inhibition EI and enzyme-substrate inhibition ESI constants for compound **4b** have been determined. The inhibitor concentration used in the kinetic experiments was 0.00, 0.0008, and 0.0016 *μ*M while concentration of substrate was 0.0, 0.1, 0.2, 0.3, and 0.4 mM. The kinetic mechanism was determined by plotting of 1/V versus 1/[S] in the presence of inhibitor concentrations which gave a series of straight lines as shown in Figure 3. The results revealed intersection of lines in the second quadrant. The kinetic results showed that maximum velocity (*V*_max_) decreased with increasing Michaelis constant (*K*_m_) as a result of increasing concentration of **4b**. The EI dissociation constant (*K*_i_) and ESI dissociation constant (*K*_i′_) for compound **4b** have also been determined from Lineweaver-Burk plots. The stronger binding of derivative **4b** with enzyme has been assured by a lower value of *K*_i_ than *K*_i′_ which also confirmed the mixed-type behaviour with a *K*_i_ value of 0.0007 *μ*M and a *K*_i′_ value of 0.0018 *μ*M, respectively (Table 2).

### 3.4. Molecular Docking Study

**Figure 4:**
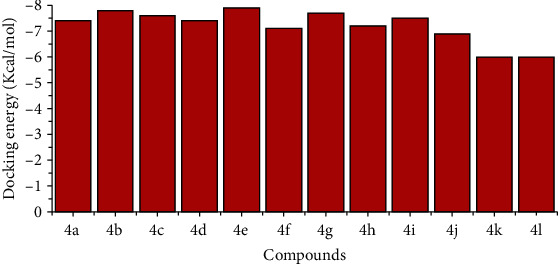
The docking energy values of synthesized compounds **4a-l**.

In order to show best conformations, derivatives (**4a–l**) were docked with target enzyme (PDBID 4H9M). The resulting complexes were observed based on the minimum energy values (kJmol^−1^) and pattern of bonding (hydrophobic and hydrophilic). Docking results justified good binding energies presented in Figure 4 with standard error—2.5 Kcal/mol. The binding energies showed the best conformations of the synthesized inhibitors in active binding sites of enzymatic protein. The derivatives **4e** and **4b** showed docking energies (−7.90 and 7.80 kJmol^−1^, respectively). Most of the docked molecules had closely related docking energies due to similar ester/amide functionalities.

#### 3.4.1. Binding Pocket Analysis of Urease-Docked Complexes

**Figure 5:**
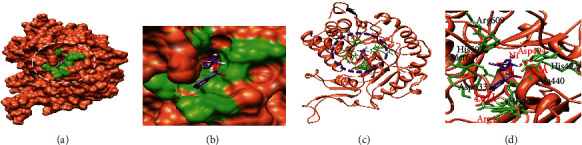
Binding interactions of compound **4b** with the active binding site of urease PDBID 4H9M generated using Discovery Studio. (a–c) Show the three-dimensional docking of derivative **4b** in a binding pocket. (d) Shows the two-dimensional ligand-protein interactions. The legend inset represents the type of interaction between the ligand atoms and the amino acid residues of the protein.

Based on the in vitro and in silico results, the **4b**-docked complex was further analyzed to learn about the structure–activity relationship (SAR) based on interactions. Docking energy values showed that docked molecules were bound in the active binding site of enzyme. The docking complexes of the most potent derivative **4b** are shown in Figure 5.

## 4. Discussion

The structures of final products **4a-l** were ascertained by FTIR spectroscopy, ^1^H, ^13^C NMR, and mass spectrometry techniques. The ^1^H NMR spectra exhibited singlet of methylene (–CH_2_–) protons at 4.7–4.9 ppm which confirmed the presence of a methylene bridge in the synthesized compounds while peak of ester carbonyl (C=O) at 164.4–164.9 ppm in the ^13^C NMR spectra confirmed successful synthesis of compounds **4a–l**. The mass spectral data of compounds **4a–l** showed molecular ion peaks according to their molecular masses with base peaks due to cleavage of ester linkages and loss of alkoxy radical (RO) and base peaks of halo benzoyl cation X–Ph–CO^+^. The final products, **4d**, **4e**, **4i**, and **4j**, containing bromine showed M and M+2 peaks with a 1.1 : 1.0 ratio according to natural abundance of bromine isotopes ^79^Br and ^81^Br in 50.5%/49.5%. The **4a**, **4b**, **4c**, **4g**, **4h**, and **4k** containing chlorine atom showed M and M+2 peaks with a ratio of 3 : 1 according to their natural isotopic abundance ^35^Cl and ^37^Cl in 75%/25%.

**Figure 6:**
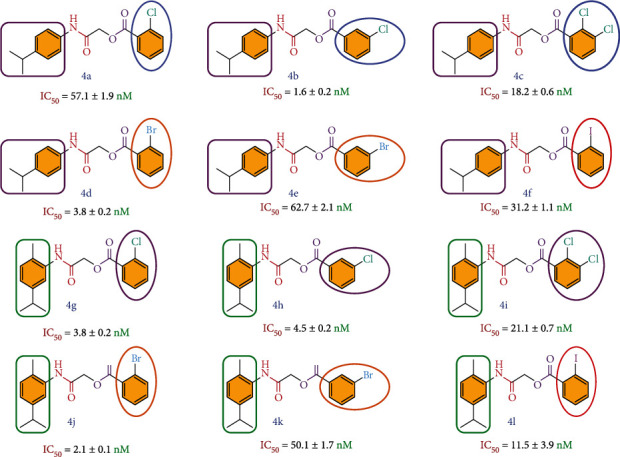
Structure–activity relationship with highlighted functional groups in **4a–l**.

The derivatives with an isopropyl-substituted phenyl ring on one side and a halogen-substituted phenyl ring on the other side showed good activity, especially compound **4b**, which exhibited the most potent inhibition with IC_50_ 1.6 nM (Figure 6). The effects on urease inhibitory activity of the chloro, bromo, iodo, and alkyl groups present on the benzene ring were also evaluated. The type and position of the functional groups at acyl core and aniline phenyl ring are the determining factors of urease inhibitory activities. The derivatives **4a** and **4b** possess the same functional groups -Cl but on a different position of the phenyl ring, thus having different activities. The derivative **4b** with 3-chloro at acyl core and 4-isopropyl group at 4-position of aniline phenyl ring is more potent than **4a**, which possesses 2-chloro at acyl core but the same aniline substitution pattern. Similarly, derivatives **4d** and **4e** have the same halogen -Br but on different positions of the benzoyl ring thus having different activities as 2-bromosubstituted derivative **4d** is more active than 3-bromosubstituted analogue **4e**. Both compounds **4d** and **4e** possess the same hydrophobic isopropyl substitution at 4-position of the aniline moiety. The presence of alkyl substitution at *ortho*- and *meta-*position of aniline also affects the urease inhibitory activity. It is evident from compounds **4g** and **4h** which possess the same -Cl at a different position of acyl core but having comparable activity. This is due to the presence of *ortho*- and *meta*-substituted aniline moiety.

The derivatives **4j** and **4k** having the same bromofunctionalities at the *ortho-* and *meta*-positions of the benzoic acid moiety and with similar aniline substitution exhibit clearly different enzyme inhibitory activities. The derivative **4j** is 25 times more active than the derivative **4k**. The iodosubstituted derivatives in general possess intermediate enzyme inhibitory activity. We may infer that halogen-substituted regioisomers along with alkyl-substituted aniline showed remarkable differences in the inhibitory activity, as in the case of *ortho-* and *meta*-chlorosubstituted derivatives **4a** and **4b** with *para*-substituted aniline. The bromosubstituted regioisomers **4d** and **4e** with *para*-substituted aniline also possess different enzyme inhibitory activities. It has been evident from the results that the derivative **4b** is more active as compared to the previously reported heterocyclic derivative **1**. The urease inhibitory activity results revealed that halo-substituted benzoic acid moiety, along with ester amide linkages, played an important role in the enzyme inhibitory activity. Different hydrophilic and hydrophobic groups were also introduced at variable positions of the phenyl ring to check their role in the inhibitory activity of jack bean urease. Alkyl substitution on aniline also influences the inhibitory activity, and in most of the cases, increasing the number of alkyl chains also increased the inhibitory activity. This indicates that the presence of hydrophobic groups also affects the inhibitory activity, showing some sort of nonpolar interaction. It also confirmed that the substitution pattern of -Cl at acyl core is not only the determining factor for the activity but the presence of alkyl substitution at aniline also play very important role in urease inhibition. Though both -Cl and -Br are present in the same group and are electron withdrawing inductively, -Cl is more electron withdrawing as compared to -Br which may be the reason of difference in urease inhibitory activity in compounds **4b** and **4e**. Another important factor which may affect the activity is the size of these atoms. -Br is larger in size compared to -Cl which may affect the enzyme inhibition. The enzyme inhibitory kinetic results revealed that inhibitor **4b** inhibits urease enzyme by the mixed type of inhibition.

The computational molecular docking results showed that docked complex of compound **4b** interacts with target protein by two hydrogen bonds. The benzyl group directly interacted with Arg439 by hydrogen bonding at a bond distance of 4.02 Å. Phenyl rings with halo substitution also showed *π*-interactions with Arg439. The carbonyl group in **4b** also forms hydrogen bond with Asp494 at 3.02 Å. The literature supports our docking results due to the presence of such functionalities [37–43]. The compound **4b** binds with the target enzyme with binding interactions which indicated that the functional groups bind with the amino acid residues present in the active binding site. There are also some interactions between functional groups of **4b** and amino acids which are present in the remote sites of the enzyme. This may suggest the allosteric binding of compound **4b** with the target enzyme. The inhibition constant (*K*_i_) value determined computationally by using the AutoDock tool was 0.916 *μ*M while the *K*_i_ value determined by in vitro studies was 0.0007 *μ*M. Based on the docking and bioassay results, it was found that **4b** may act as a lead structure to discover clinical jack bean urease inhibitor.

## 5. Conclusion

The jack bean urease inhibitors **4a–l**, having excellent urease inhibitory potential than the standard thiourea, were described in the present work. A series of compounds, **4a–l**, having ester/amide functionalities, were synthesized in order to check their inhibitory potential against jack bean urease. Simple synthetic routes were adopted to synthesize the desired compounds in good yield. The inhibitory activity results showed that compound **4b**, having a chloro group at the *meta*-position of acyl core and *para*-alkyl-substituted aniline, showed excellent inhibitory potential against urease enzyme with an IC_50_ of 1.6 ± 0.2 nM, much better than the standard thiourea with an IC_50_ of 472.1 ± 135.1 nM. The presence of a 2-chlorosubstituted phenyl ring and a 4-isopropyl-substituted aniline on the other side, in the case of compound **4b**, played a vital role in urease inhibitory activity. The bioassay results confirmed that the substitution pattern of -Cl at the acyl core is not only the determining factor for activity but alkyl substitution at aniline also play very important role in urease inhibition. In the kinetic studies, a mixed-type inhibition mechanism was reflected in the Lineweaver–Burk plots for compound **4b**. The molecular docking studies showed that the predicted binding affinities of the synthesized compounds are excellent, especially in the case of compounds **4e** and **4b**, having energies of −7.9 and − 7.8 Kcal/mol, respectively. It can be concluded from our results that compound **4b** is a highly potent urease inhibitor.

## Figures and Tables

**Table 1:** Urease inhibition (IC_50_) values of synthesized derivatives **4a–l.**

Compound	Urease (jack bean) inhibition IC_50_ ± SD (nM)
4a	57.1 ± 1.9
4b	1.6 ± 0.2
4c	18.2 ± 0.6
4d	3.8 ± 0.2
4e	62.7 ± 2.1
4f	31.2 ± 1.1
4g	3.8 ± 0.2
4h	4.5 ± 0.2
4i	21.1 ± 0.7
4j	2.1 ± 0.1
4k	50.1 ± 1.7
4l	11.5 ± 3.9
Thiourea	472.1 ± 135.1

**Table 2:** Kinetic parameters of the jack bean urease for urea activity in the presence of different concentrations of compound **4b.**

Code	Dose (*μ*M)	1/*V*_max_ (*Δ*A/min)	*K*_m_ (mM)	Inhibition type	*K*_i_ (*μ*M)	*K*_i′_ (*μ*M)
**4b**	0.00	0.001796	3.076	Mixed inhibition	0.0007	0.0018
	0.0008	0.00099	5.263			
	0.0016	0.00088	5.555			

## Data Availability

Date could be provided upon request from Dr. Zaman Ashraf.
